# An Activation Likelihood Estimation Meta-Analysis of How Language Balance Impacts the Neural Basis of Bilingual Language Control

**DOI:** 10.3390/brainsci15080803

**Published:** 2025-07-28

**Authors:** Tao Wang, Keyi Yin, Qi Zhou, Haibo Hu, Shengdong Chen, Man Wang

**Affiliations:** 1 School of Psychology, Qufu Normal University, Jining 273165, China; qfnuwangtao@qfnu.edu.cn (T.W.); keyiyin@qfnu.edu.cn (K.Y.); zhouqi@qfnu.edu.cn (Q.Z.); huhaibo611420@qfnu.edu.cn (H.H.); 2 School of Foreign Languages, Qingdao University, Qingdao 266071, China

**Keywords:** bilingual language control, language balance, fMRI, meta-analysis

## Abstract

**Background:** Neurological networks involved in bilingual language control have been extensively investigated. Among the factors that influence bilingual language control, language balance has recently been proposed as a critical one. Nevertheless, it remains understudied how the neural basis of bilingual language control is affected by language balance. **Methods:** To address this gap, we conducted a meta-analysis of functional magnetic resonance imaging (fMRI) studies on bilingual language control using Ginger ALE, with language balance as a moderating factor. **Results:** Conjunction analyses revealed a domain-general pattern of neural activities shared by balanced and unbalanced bilinguals, with convergent activation observed in the left precentral gyrus and left medial frontal gyrus. Regarding domain-specificity, contrast analyses did not identify stronger activation convergence in balanced bilinguals compared to unbalanced bilinguals. However, unbalanced bilinguals exhibited significantly stronger convergence of activation in the left middle frontal gyrus, left inferior frontal gyrus, and left precuneus. **Conclusions:** These findings suggest that language balance can modify the neural mechanisms of bilingual language control, with unbalanced bilinguals relying on more domain-general cognitive control resources during bilingual language control.

## 1. Introduction

In recent years, bilingual language control—the cognitive mechanism to enable bilingual individuals to select their target language while minimizing interference from the non-selected language—has garnered increasing attention [1,2,3]. Empirical evidence suggests that language balance may influence the bilingual language control process [4,5,6,7]. Language balance has been defined as a balanced level of proficiency in two languages [7]. Accordingly, balanced bilinguals are highly proficient in both languages, whereas unbalanced bilinguals typically exhibit dominance in one language over the other. Based on the inhibitory control model [8], we argue that unbalanced bilinguals, due to the greater activation of their dominant language, require stronger inhibition to suppress the dominant language and produce the weaker one successfully. In contrast to unbalanced bilinguals, balanced bilinguals exhibit more automaticity in selecting the target language, relying less on inhibitory control, and engaging more with language-specific lexical selection mechanisms [8,9].

Previous empirical studies have found differences in the neural basis of bilingual language control between balanced and unbalanced bilinguals, but there are still disputes over whether the differences lie in neural activation patterns [4,10] or only in activation intensity within the same neural basis [11]. Furthermore, due to the limited sample sizes and the language pairs being tested (e.g., Spanish-French or Chinese-English), previous neuroimaging studies on bilingual language control are restricted in their generalizability to diverse bilingual populations [12].

Therefore, this study employed the meta-analytic technique to examine the neural basis of bilingual language control in balanced bilinguals and unbalanced bilinguals. Specifically, the Activation Likelihood Estimation (ALE) technique was chosen since it’s a widely used meta-analytic technique for neuroimaging data, especially on bilingual language control [6,13,14,15].

### 1.1. Bilingual Language Control

The inhibitory control model suggests that bilinguals are required to suppress the unused language during communication to minimize interference from the non-target language, thereby producing accurate output [8]. Learning and using a second language (L2) heightens interference, thereby imposing greater demands on mechanisms for controlling interference [8,10]. The adaptive control hypothesis extends the inhibitory control model by proposing that bilinguals’ interference control skills are enhanced as their L2 proficiency develops [16]. Therefore, based on these models, unbalanced bilinguals require greater cognitive resources for managing interference during language switching compared to balanced bilinguals.

The language switching task is frequently employed to investigate bilingual language control. This task requires bilinguals to alternate between languages (switch trials) or remain in the same language (repeat trials). The language switching cost, calculated as the difference in response times (RTs) and/or accuracy between switch- and repeat-trials, serves as a psychological index of bilingual language control [8]. Functional magnetic resonance imaging (fMRI) studies have also used the language switching task to identify the neural mechanisms underlying bilingual language control [17,18].

Neuroimaging studies have revealed that bilinguals recruit brain regions associated with cognitive control to select the target language and suppress interference from the non-targeted language, including the bilateral dorsolateral prefrontal cortex and the bilateral inferior frontal gyrus [19,20,21,22]. In a review of language switching studies, ref. [10] proposed a bilingual language control network. This network integrates cortical and subcortical regions, such as the anterior cingulate cortex, basal ganglia, inferior parietal gyrus, and the prefrontal cortex. Each region performs distinct cognitive control functions, including language planning, representation maintenance, set switching, conflict monitoring, response inhibition, and lexical selection.

### 1.2. Language Balance

Balanced bilinguals demonstrate greater efficiency in bilingual language control and exhibit enhanced cognitive advantages compared to unbalanced bilinguals [7]. The adaptive control hypothesis proposes that as L2 proficiency increases, bilinguals’ ability to manage interference is enhanced [16]. This point is supported by numerous neuroimaging studies demonstrating similar cortical activation in balanced bilinguals when using both languages, while unbalanced bilinguals exhibit additional frontal activation linked to domain-general cognitive control [4,10]. Reduced activation in the frontal regions among balanced bilinguals indicates improved efficiency in information processing, aligning with the principle that greater proficiency reduces cognitive effort [23]. Therefore, it is reasonable to conclude that language balance has a significant effect on the neural basis of bilingual language control.

Nevertheless, previous research did not reach a consensus regarding the definition and measurement of language balance. Ref. [24] argued that language balance should reflect equivalent performance across grammatical, lexical, and pragmatic dimensions of the two languages. In contrast, ref. [25] argued that the language balance should place more emphasis on the social functions and situational needs of language use. At the core of this debate lies the question of whether language balance should be measured in terms of linguistic symmetry or functional adaptability.

To address this issue, we propose the concept of relative language balance, which refers to bilingual ability as a dynamic state that adapts to specific contexts, tasks, and individual needs. This concept is in accordance with the Dynamic Systems Theory [16,26], which suggests that bilingual performance may evolve over time and across environments. Bilinguals achieve relative language balance when their linguistic abilities are sufficiently adaptable to meet situational demands. This concept accommodates both linguistic symmetry and functional adaptability, avoiding an overly simplistic definition of language balance. Empirical evidence supports this perspective, as bilinguals often demonstrate flexible resource allocation across languages in cognitively demanding tasks [27], aligning with the hypothesis of relative language balance.

### 1.3. The Present Study

This meta-analysis examined balanced and unbalanced bilinguals, categorized by self-assessed proficiency differences in L1 and L2 [28]. Separate ALE meta-analyses were conducted for balanced and unbalanced bilinguals during language switching tasks to identify unique and shared brain activation patterns. Conjunction and contrast analyses were then performed to assess neural commonalities and differences across groups. By this means, we hope to contribute to the understanding of the neural basis of bilingual language control of balanced and unbalanced bilinguals, especially whether the differences between these two groups are reflected in the activation pattern or the activation intensity.

## 2. Materials and Methods

### 2.1. Literature Search and Exclusion Criteria

This meta-analysis was conducted using the Preferred Reporting Items for Systematic Reviews and Meta-Analysis (PRISMA) statement guidelines (http://www.prisma-statement.org/ (accessed on 16 December 2024)) [29], as well as more recent guidelines for conducting systematic reviews [30,31]. Notably, our meta-analyses were not preregistered. We conducted a literature search on the Web of Science and PubMed databases using the terms “bilingual language control” combined with “functional magnetic resonance imaging (fMRI)”, “Positron Emission Tomography (PET)”, “brain imaging”, or “neuroimaging” up to December 2024. This preliminary search yielded a total of 1972 records from both platforms; 750 articles remained after removing duplicates (n = 1222). Six additional studies [32,33,34,35,36,37] were identified through complementary search methods, namely backward reference searching (checking reference lists in published articles) [14], resulting in 756 records for initial screening. Two authors independently conducted the first round of screening based on titles and abstracts, using the following inclusion criteria: (1) fMRI or PET studies; (2) studies that recruited healthy adults aged 18–50; (3) peer-reviewed journal articles (excluding conference papers or abstracts). A total of 145 articles passed the first round of screening and were then read in full to confirm their eligibility. In the second round of screening, studies containing any of the following characteristics were excluded from further analyses: (1) did not use fMRI or PET techniques (n = 11); (2) not primarily focused on bilingual language control (n = 55); (3) did not report whole-brain activation (n = 15); (4) did not report peak coordinates in Talairach or Montreal Neurological Institute (MNI) stereotaxic space (n = 25); (5) involving bimodal bilinguals (i.e., one of the languages is sign language; n = 6); (6) involving language comprehension studies (n = 3). The exclusion criterion 6 was made because the control processes involved in language production and comprehension are not entirely the same [38,39]. (7) examined bilingual language control performance in relatively extreme contexts, namely interpreting or simultaneous translation (n = 5). The exclusion criterion 7 was made because these studies often investigate the mechanisms behind professional performance or specific strategies behind translation, rather than bilingual language control itself; and the type of bilingual language control required during interpreting and translation is different from the typical bilingual language control required by bilinguals in everyday language use environments [14].

**Figure 1:**
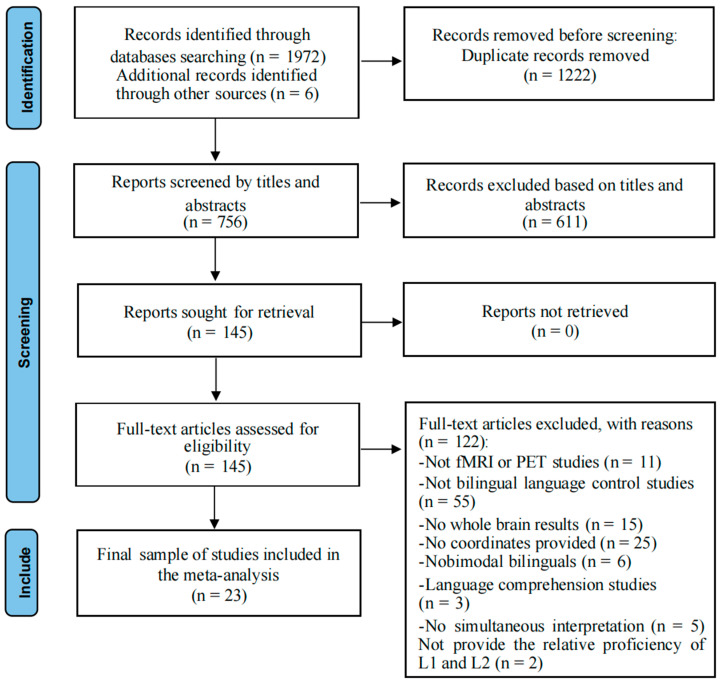
PRISMA flow diagram of the literature search for the meta-analysis.

We were confident in including different language tasks from different languages into the same analysis because the algorithm we used searches for converging areas of activation between different experiments, thus only providing consistently replicated activations. Furthermore, the participants in these studies were comparable in terms of gender and age (typically young adults), which ensured relatively robust findings. This study compiled a total of 23 neuroimaging studies, all of which investigated bilingual language control (see Figure 1, Table 1). Language switching, a common task in bilingual language control research [2], was included as an experimental condition in all these studies. Additionally, each participant completed a baseline task similar to the experimental condition but without requiring language switching before the experiment. Since most of the studies (17 out of 23) included in our meta-analysis did not distinguish switching directions, our analysis combined the results of switches from L1 to L2 and from L2 to L1 (see Table S1).

### 2.2. Coding of Study Characteristics

The following variables were coded for each study: (1) number of participants; (2) age of participants; (3) L1 and L2; (4) task; (5) contrast; (6) AOA; (7) L1/L2 proficiency ratio; (8) the number of foci.

### 2.3. Data Analysis

We performed coordinate-based meta-analyses using Ginger ALE 3.0.2 (BrainMap.org) to examine language-switching-related brain activation in balanced bilinguals (n = 88 coordinates) and unbalanced bilinguals (n = 257 coordinates). All analyses employed the non-additive ALE algorithm [56] to minimize within-experiment effects and enable population-level inference [57]. Coordinates originally reported in Talairach space were transformed to MNI space using the “Convert Foci” tool in Ginger ALE [58]. For individual group analyses, we applied a recommended cluster-forming threshold of *p* < 0.001 and a cluster-level threshold of *p* < 0.05 [59,60]. To identify shared and distinct neural bases between balanced and unbalanced bilinguals during language switching, we conducted conjunction and contrast analyses with an uncorrected threshold of *p* < 0.01, 5000 permutations, and a minimum cluster size of 100 mm^3^, following established meta-analytic procedures [14]. Visualization of the results was performed using Mango 4.1 software (http://mangoviewer.com/mango.html (accessed on 20 January 2025)).

## 3. Results

### 3.1. Language Switching Costs in Balanced Bilinguals

**Figure 2:**
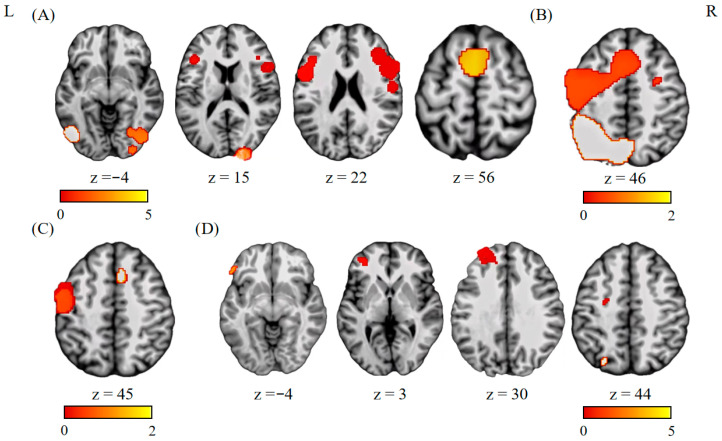
Results of functional imaging meta-analyses. (**A**) Single-dataset analysis of studies on balanced bilinguals. (**B**) Single-dataset analysis of studies on unbalanced bilinguals. (**C**) Conjunction analysis between studies of balanced and unbalanced bilinguals. (**D**) Contrast analysis between balanced bilinguals and unbalanced bilinguals (unbalanced bilinguals > balanced bilinguals).

This analysis included six experiments, 110 participants, and 88 foci. In the left hemisphere, we found consistent activations in the precentral gyrus (BA 6), medial frontal gyrus (BA 6), and Fusiform (BA 19). In the right hemisphere, activation clusters were identified in the middle frontal gyrus (BA 9) and cuneus (BA 17) (see Figure 2A and Table 2).

### 3.2. Language Switching Costs in Unbalanced Bilinguals

This analysis included 17 experiments, 425 participants, and 257 foci. In the left hemisphere, we found consistent activations in the superior frontal gyrus (BA 6) and inferior parietal lobe (BA 40) (see Figure 2B and Table 2).

### 3.3. Conjunction Analysis

The conjunction analysis between the balanced and unbalanced bilinguals in the language switching costs showed convergence of activation in the left precentral gyrus (BA 6) and left medial frontal gyrus (BA 6) (see Figure 2C and Table 3).

### 3.4. Contrast Analyses

The contrast between balanced and unbalanced bilinguals revealed no significant activation. The contrast between unbalanced and balanced bilinguals revealed significant activation in clusters of the left middle frontal gyrus (BA 6, 9, 10), left inferior frontal gyrus (BA 45), and the left precuneus (BA 7) (see Figure 2D and Table 4).

## 4. Discussion

The present study investigated the neural basis of bilingual language control in bilinguals, focusing on how language balance influences this process. Drawing upon the inhibitory control model and adaptive control hypothesis, this meta-analysis systematically examined brain activation patterns during language switching tasks in balanced and unbalanced bilinguals. The conjunction analysis identified two commonly activated brain regions across both bilingual groups: the left precentral gyrus and the left medial frontal gyrus. Contrast analyses revealed that compared with balanced bilinguals, unbalanced bilinguals exhibited significantly enhanced activation in the left middle frontal gyrus, left inferior frontal gyrus, and left precuneus during language switching. These findings suggest that while frontal regions are crucial for language switching in all bilinguals, unbalanced bilinguals require additional recruitment of brain regions. Previous research has demonstrated that these brain regions are also activated during non-linguistic executive control tasks [61,62], indicating that unbalanced bilinguals rely more on domain-general cognitive control resources during language switching.

### 4.1. Commonalities in Brain Regions Associated with Balanced and Unbalanced Bilinguals’ Language Switching

Conjunction analysis results revealed that both balanced and unbalanced bilinguals activated the left precentral gyrus and the left medial frontal gyrus during language switching. The left precentral gyrus has been linked to phonological retrieval [63,64,65]. Ref. [1] found that, compared to monolingual naming, the left precentral gyrus showed significant activation in bilingual environments. A meta-analysis study by [66] reported that the left precentral gyrus is involved in monitoring language working memory but not in processing lexical–semantic information.

Another key component of the bilingual language control network identified in this meta-analysis is the left medial frontal gyrus. The left medial frontal gyrus is closely associated with cognitive control during bilingual language production [67]. This region overlaps with the dACC/pre-supplementary motor area that is often activated by bilingual language switching [12] and represents a key part of the domain-general conflict monitoring network [68,69]. The results of our study indicate that, regardless of the language balance of the two types of bilinguals, the left precentral gyrus and the left medial frontal gyrus are activated during bilingual language control. Our study further confirms the importance of the left precentral gyrus and the left medial frontal gyrus for successfully achieving bilingual language control through meta-analysis.

### 4.2. Differences in Brain Regions Associated with Balanced and Unbalanced Bilinguals’ Language Switching

Contrast analysis identified distinct patterns of brain activation between balanced and unbalanced bilinguals during language switching. Compared to balanced bilinguals, unbalanced bilinguals exhibited significantly greater activation in the left middle frontal gyrus, left inferior frontal gyrus, and left precuneus during language switching. The left middle frontal gyrus plays a central role in bilingual cognitive control and is traditionally thought to be associated with executive control processes such as response selection, task switching, and the inhibition of irrelevant items in working memory [61,70,71,72]. Additionally, a previous fMRI study on language switching in unbalanced bilinguals confirmed the involvement of the left middle frontal gyrus in interference control [49].

The left inferior frontal gyrus is associated with response inhibition [73] as well as the selection of competing options [74,75]. This brain region is activated during interference control [76,77]. For example, ref. [77] conducted a rapid response task to investigate how Spanish monolinguals and Spanish–Catalan bilinguals manage interference from the non-target language. The results revealed that, compared to Spanish monolinguals, Spanish–Catalan bilinguals exhibited greater activation in the left inferior frontal gyrus, suggesting that higher activation in this area facilitates the control of lexical interference. The left inferior frontal gyrus is also involved in general executive functions [62,78], as demonstrated by the Stroop and Flanker tasks, both of which are indicators of domain-general cognitive control [79]. For instance, ref. [80] found that in the Flanker task, variations in left inferior frontal gyrus activation were associated with behavioral performance during interference suppression and response inhibition.

The precuneus serves as a functional core of the default mode network. Its broad connections facilitate the integration of internally and externally driven information [81]. During cognitively demanding tasks, precuneus activity increases [81,82]. For example, ref. [42] conducted an fMRI study in which German–English bilinguals performed bilingual language control tasks prompted in German or English. The results revealed stronger activation in the left precuneus during switch trials compared to repetition trials. The left precuneus is also critical to the frontoparietal control network, which is linked to executive functions and facilitates processes such as attention, working memory [83,84], and general intelligence [85,86].

In summary, our findings provide evidence supporting the inhibitory control model [8] and the adaptive control hypothesis [16]. Both theories propose that bilinguals require the inhibition of the non-target language during language production to achieve accurate output in the target language, a process that relies on top-down cognitive control. As bilingual proficiency increases, the cognitive effort necessary to suppress the non-target language gradually decreases. Our findings align with this perspective, suggesting that compared to balanced bilinguals, unbalanced bilinguals encounter greater demands for interference control during language switching, as they generally require increased top-down cognitive control to suppress irrelevant linguistic information. This is evidenced by the significantly heightened activation of the left middle frontal gyrus, left inferior frontal gyrus, and left precuneus in unbalanced bilinguals. The specific activation of these brain regions highlights the neural mechanisms employed by unbalanced bilinguals to resolve interference and detect conflicts during bilingual language control. In conclusion, these findings offer novel insights into how language balance influences the neural mechanisms underlying bilingual language control. Specifically, compared to balanced bilinguals, unbalanced bilinguals must recruit additional brain regions associated with executive control to effectively manage two languages.

### 4.3. Limitations and Future Directions

Firstly, we employed self-assessed proficiency differences between L1 and L2 to differentiate balanced and unbalanced bilinguals. Although this method provides a relatively objective means of assessing language balance, as previously noted, language balance is a flexible response to situational demands and constitutes a dynamic, experience-dependent construct grounded in Dynamic Systems Theory. Future meta-analyses could adopt more rigorous classification methods for language balance based on specific research objectives, which may further elucidate the neural basis of bilingual language control in these two groups.

Secondly, our analyses included more studies on unbalanced bilinguals than on balanced bilinguals. This imbalance in sample size may introduce bias into the neural contrasts derived from the analyses. To the best of our knowledge, however, there is currently no established method to assess the potential bias introduced by such imbalances. Nevertheless, it is important to note that the disparity in the number of studies included in the current meta-analysis falls within the range of differences reported in previous bilingual language control meta-analyses, which supports the reliability of the ALE findings [3].

Thirdly, ref. [87] suggested that the direction of language switching might influence bilingual control mechanisms. For instance, in unbalanced bilinguals, switching from L2 to L1 typically incurs greater switch costs than switching from L1 to L2. While the present study identified both commonalities and differences in the neural correlates of bilingual language control between the two groups and discussed their potential functional roles, the extent to which these overlapping and distinct regions are influenced by switching direction remains unclear. Future studies should consider both bilingual proficiency and switching direction when investigating the neural mechanisms of bilingual language control.

## 5. Conclusions

The results of this meta-analysis showed that the left middle frontal gyrus, left inferior frontal gyrus, and left precuneus during language switching were more strongly activated among unbalanced than balanced bilinguals during bilingual language control. This suggests that language balance can modify the neural mechanisms of bilingual language control. Given that unbalanced bilinguals often face greater challenges in managing two languages due to their unequal proficiency levels, our findings imply that they may rely on more domain-general cognitive control resources during bilingual language control than balanced bilinguals.

## Figures and Tables

**Table 1:** A meta-analysis of the language conversion studies of balanced and unbalanced bilinguals.

	Participants (Female)	Mean Age	L1	L2	Task	Contrast	AoA	L1/L2 Proficiency Ratio	Foci
Balanced bilinguals (n = 6)									
Garbin [33]	19 (12)	20.3	Spanish	Catalan	Picture naming	Switch > non-switch	<4	1	35
Köpke [40]	20 (8)	28.7	English/ French	English/ French	Picture naming	Switch > baseline	0/11	1.05	27
Price [41]	6 (0)	30.5	German	English	Reading task	Switch > non-switch	8.8	0.93	3
Reverberi [42]	21 (12)	23.1	German	English	Picture naming	Switch > non-switch	9.1	1.15	8
Stasenko [21]	24 (18)	20	Spanish	English	Reading task	Switch > non-switch	4.1	0.94	5
Weissberger [36]	20 (15)	21	Spanish	English	Digit naming	Switch > baseline	5.1	0.93	10
Unbalanced bilinguals (n = 17)									
Fu [43]	21 (13)	22	Chinese	English	Picture naming	Switch > non-switch	10	1.43	4
Geng [20]	19 (10)	23.21	Chinese	English	Picture naming	Switch > non-switch	8	1.71	18
Guo [18]	24 (12)	22.1	Chinese	English	Picture naming	Switch > non-switch	12	1.40	44
Hernandez [44]	6 (4)	21.7	Spanish	English	Picture naming	Switch > non-switch	<5	0.65	3
Hernandez [45]	12 (5)	21.4	Spanish	English	Picture naming	Switch > non-switch	5	0.68	4
Lehtonen [46]	11 (10)	31.8	Finnish	Norwegian	Translation task	Switch > non-switch	26.7	1.26	2
Liu [47]	39 (29)	22.1	Chinese	English	Picture naming	Switch > non-switch	12.8	1.54	24
Ma [48]	22 (5)	22.6	Chinese	English	Picture naming	Switch > baseline	12	-	10
Wang [49]	12 (6)	19.5	Chinese	English	Picture naming	Switch > non-switch	12.67	1.54	15
Wang [50]	15 (8)	20.5	Chinese	English	Digit naming	Switch > non-switch	12.06	1.41	24
Wang [51]	69 (35)	22.8	Chinese	English	Picture naming	Switch > non-switch	6	1.57	5
Wu [52]	59 (35)	22.3	Chinese	English	Picture naming	Switch > non-switch	9.33	1.51	10
Yuan [53]	32 (21)	22.3	Chinese	English	Picture naming	Switch > non-switch	9.78	1.41	8
Zhang [54]	21 (9)	24.38	Chinese	English	Speech production task	Switch > non-switch	12.4	1.37	20
Zhang [55]	16 (9)	21.3	Chinese	English	Digit naming	Switch > non-switch	11.9	1.08	15
Zhang [37]	22 (12)	19.9	Chinese	English	Picture naming	Switch > non-switch	11.6	1.29	34
Zhang [22]	25 (14)	20.2	Chinese	English	Picture naming	Switch > non-switch	10.1	1.41	17

**Table 2:** Results of the meta-analysis on language switching costs of balanced bilinguals studies and unbalanced bilinguals studies.

Cluster	Region	L/R	MNI Coordinates			Volume (mm^3^)	Peak ALE Value
			x	y	z		
Language Switching Costs of Balanced Bilinguals							
1	Mid Frontal Gyrus (BA 9)	R	38	28	28	24,661	0.0015
2	Precentral Gyrus (BA 6)	L	−50	−6	38	22,101	0.0020
3	Cuneus (BA 17)	R	20	−96	10	21,897	0.0016
4	Med Frontal Gyrus (BA 6)	L	0	4	60	19,957	0.0016
5	Fusiform Gyrus (BA 19)	L	−46	−70	−6	11,855	0.0014
Language Switching Costs of Unbalanced Bilinguals							
1	Sup Frontal Gyrus (BA 6)	L	−4	4	66	128,841	0.0055
2	Inf Parietal Lobe (BA 40)	L	−38	−50	48	40,499	0.0041

**Table 3:** Conjunction analysis between language switching costs of balanced bilinguals and of unbalanced bilinguals.

Cluster	Region	L/R	MNI Coordinates			Volume (mm^3^)	Peak ALE Value
			x	y	z		
1	Precentral Gyrus (BA 6)	L	−50	−6	38	16,837	0.0020
2	Med Frontal Gyrus (BA 6)	L	0	4	60	15,807	0.0016

**Table 4:** Contrast analysis between language switching costs of balanced bilinguals and of unbalanced bilinguals (unbalanced bilinguals > balanced bilinguals).

Cluster	Region	L/R	MNI Coordinates			Volume (mm^3^)	Z
			x	y	z		
1	Mid Frontal Gyrus (BA 9)	L	−40	51	24	8016	2.3495
2	Mid Frontal Gyrus (BA 6)	L	−24	−10	46	2527	2.7065
3	Inf Frontal Gyrus (BA 45)	L	−52	28	−3	787	2.3263
4	Mid Frontal Gyrus (BA 10)	L	−37.5	47	4.7	577	2.3416
5	Precuneus Gyrus (BA 7)	L	−27	−67	43	333	2.3656

## Data Availability

No new data were created in this meta-analysis. Coordinate files and result maps from the meta-analysis are available upon request.

## Supplementary Materials

The following supporting information can be downloaded at: https://www.mdpi.com/article/10.3390/brainsci15080803/s1, Table S1: A Meta-Analysis of the Language Conversion Studies of Balanced and Unbalanced Bilinguals.

## References

[1] Abutalebi J., Annoni J.M., Zimine I., Pegna A.J., Seghier M.L., Lee-Jahnke H., Khateb A. (2008). Language control and lexical competition in bilinguals: An event-related fMRI study. Cereb. Cortex.

[2] Declerck M., Philipp A.M. (2015). A review of control processes and their locus in language switching. Psychon. Bull. Rev..

[3] Jiao L., Meng N., Wang Z., Schwieter J.W., Liu C. (2022). Partially shared neural mechanisms of language control and executive control in bilinguals: Meta-analytic comparisons of language and task switching studies. Neuropsychologia.

[4] Abutalebi J. (2008). Neural aspects of second language representation and language control. Acta Psychol..

[5] Cargnelutti E., Tomasino B., Fabbro F. (2019). Language brain representation in bilinguals with different age of appropriation and proficiency of the second language: A meta-analysis of functional imaging studies. Front. Hum. Neurosci..

[6] Sulpizio S., Del Maschio N., Fedeli D., Abutalebi J. (2020). Bilingual language processing: A meta-analysis of functional neuroimaging studies. Neurosci. Biobehav. Rev..

[7] Yow W.Q., Li X. (2015). Balanced bilingualism and early age of second language acquisition as the underlying mechanisms of a bilingual executive control advantage: Why variations in bilingual experiences matter. Front. Psychol..

[8] Green D.W. (1998). Mental control of the bilingual lexico-semantic system. Biling. Lang. Cogn..

[9] Costa A., Santesteban M. (2004). Lexical access in bilingual speech production: Evidence from language switching in highly proficient bilinguals and L2 learners. J. Mem. Lang..

[10] Abutalebi J., Green D. (2007). Bilingual language production: The neurocognition of language representation and control. J. Neurolinguist..

[11] Mouthon M., Khateb A., Lazeyras F., Pegna A.J., Lee-Jahnke H., Lehr C., Annoni J.M. (2020). Second-language proficiency modulates the brain language control network in bilingual translators: An event-related fMRI study. Biling. Lang. Cogn..

[12] Luk G., Green D.W., Abutalebi J., Grady C. (2012). Cognitive control for language switching in bilinguals: A quantitative meta-analysis of functional neuroimaging studies. Lang. Cogn. Process..

[13] Eickhoff S.B., Bzdok D., Laird A.R., Kurth F., Fox P.T. (2012). Activation likelihood estimation meta-analysis revisited. Neuroimage.

[14] Tao L., Wang G., Zhu M., Cai Q. (2021). Bilingualism and domain-general cognitive functions from a neural perspective: A systematic review. Neurosci. Biobehav. Rev..

[15] Turkeltaub P.E., Eden G.F., Jones K.M., Zeffiro T.A. (2002). Meta-analysis of the functional neuroanatomy of single-word reading: Method and validation. Neuroimage.

[16] Green D.W., Abutalebi J. (2013). Language control in bilinguals: The adaptive control hypothesis. J. Cogn. Psychol..

[17] De Bruin A., Roelofs A., Dijkstra T., FitzPatrick I. (2014). Domain-general inhibition areas of the brain are involved in language switching: FMRI evidence from trilingual speakers. NeuroImage.

[18] Guo T., Liu H., Misra M., Kroll J.F. (2011). Local and global inhibition in bilingual word production: FMRI evidence from Chinese–English bilinguals. NeuroImage.

[19] Coderre E.L., Smith J.F., Van Heuven W.J.B., Horwitz B. (2016). The functional overlap of executive control and language processing in bilinguals. Biling. Lang. Cogn..

[20] Geng L., Zhao X., Xu Q., Wu H., Hu X., Liu Z., Ming L., Xue Z., Yue C., Yang Y. (2024). Cognitive and neural mechanisms of voluntary versus forced language switching in Chinese–English bilinguals: An fMRI study. Cereb. Cortex.

[21] Stasenko A., Hays C., Wierenga C.E., Gollan T.H. (2020). Cognitive control regions are recruited in bilinguals’ silent reading of mixed-language paragraphs. Brain Lang..

[22] Zhang Y., Zhao J., Huang H., Zhang Z., Wu S., Qiu J., Wu Y.J. (2024). Neuroimaging evidence dissociates forced and free language selection during bilingual speech production. Biling. Lang. Cogn..

[23] Bialystok E., Poarch G. (2014). Language experience changes language and cognitive ability. Z. Erziehungswiss..

[24] Grosjean F. (1989). Neurolinguists, beware! The bilingual is not two monolinguals in one person. Brain Lang..

[25] Wei L. (2000). The Bilingualism Reader.

[26] Herdina P., Jessner U. (2002). A Dynamic Model of Multilingualism: Perspectives of Change in Psycholinguistics.

[27] Bialystok E. (2017). The bilingual adaptation: How minds accommodate experience. Psychol. Bull..

[28] Archila-Suerte P., Woods E.A., Chiarello C., Hernandez A.E. (2018). Neuroanatomical profiles of bilingual children. Dev. Sci..

[29] Moher D., Liberati A., Tetzlaff J., Altman D.G. (2009). Preferred reporting items for systematic reviews and meta-analyses: The PRISMA statement. Ann. Intern. Med..

[30] Harari M.B., Parola H.R., Hartwell C.J., Riegelman A. (2020). Literature searches in systematic reviews and meta-analyses: A review, evaluation, and recommendations. J. Vocat. Behav..

[31] Siddaway A.P., Wood A.M., Hedges L.V. (2019). How to do a systematic review: A best practice guide for conducting and reporting narrative reviews, meta-analyses, and meta-syntheses. Annu. Rev. Psychol..

[32] Bartolotti J., Bradley K., Hernandez A.E., Marian V. (2017). Neural signatures of second language learning and control. Neuropsychologia.

[33] Garbin G., Costa A., Sanjuan A., Forn C., Rodriguez-Pujadas A., Ventura N., Belloch V., Hernandez M., Ávila C. (2011). Neural bases of language switching in high and early proficient bilinguals. Brain Lang..

[34] Hosoda C., Hanakawa T., Nariai T., Ohno K., Honda M. (2012). Neural mechanisms of language switch. J. Neurolinguist..

[35] Marian V., Bartolotti J., Rochanavibhata S., Bradley K., Hernandez A.E. (2017). Bilingual cortical control of between- and within-language competition. Sci. Rep..

[36] Weissberger G.H., Gollan T.H., Bondi M.W., Clark L.R., Wierenga C.E. (2015). Language and task switching in the bilingual brain: Bilinguals are staying, not switching, experts. Neuropsychologia.

[37] Zhang Y., Wang K., Yue C., Gao S., Huang P., Wang T., Wen X., Qiu J., Wu Y.J. (2019). Prefrontal sensitivity to changes in language form and semantic content during speech production. Brain Lang..

[38] Ahn D., Abbott M.J., Rayner K., Ferreira V.S., Gollan T.H. (2020). Minimal overlap in language control across production and comprehension: Evidence from read-aloud versus eye-tracking tasks. J. Neurolinguist..

[39] Li C., Midgley K.J., Ferreira V.S., Holcomb P.J., Gollan T.H. (2024). Different language control mechanisms in comprehension and production: Evidence from paragraph reading. Brain Lang..

[40] Köpke B., Howells R.K., Cortelazzo F., Péran P., de Boissezon X., Lubrano V. (2021). Functional and structural differences in brain networks involved in language processing and control in highly proficient early and late bilinguals. J. Neurolinguist..

[41] Price C.J., Green D.W., Von Studnitz R. (1999). A functional imaging study of translation and language switching. Brain.

[42] Reverberi C., Kuhlen A., Abutalebi J., Greulich R.S., Costa A., Seyed-Allaei S., Haynes J.D. (2015). Language control in bilinguals: Intention to speak vs. execution of speech. Brain Lang..

[43] Fu Y., Lu D., Kang C., Wu J., Ma F., Ding G., Guo T. (2017). Neural correlates for naming disadvantage of the dominant language in bilingual word production. Brain Lang..

[44] Hernandez A.E., Dapretto M., Mazziotta J., Bookheimer S. (2001). Language switching and language representation in Spanish-English bilinguals: An fMRI study. NeuroImage.

[45] Hernandez A.E. (2009). Language switching in the bilingual brain: What’s next?. Brain Lang..

[46] Lehtonen M., Laine M., Niemi J., Thomsen T., Vorobyev V.A., Hugdahl K. (2005). Brain correlates of sentence translation in Finnish-Norwegian bilinguals. NeuroReport.

[47] Liu H., Guo Z., Jiang Y., Schwieter J.W., Wang F. (2023). Neural circuits underlying language control and modality control in bilinguals: An fMRI study. Neuropsychologia.

[48] Ma H., Hu J., Xi J., Shen W., Ge J., Geng F., Wu Y., Guo J., Yao D. (2014). Bilingual cognitive control in language switching: An fMRI study of English-Chinese late bilinguals. PLoS ONE.

[49] Wang Y., Xue G., Chen C., Xue F., Dong Q. (2007). Neural bases of asymmetric language switching in second-language learners: An er-fMRI study. NeuroImage.

[50] Wang Y., Kuhl P.K., Chen C., Dong Q. (2009). Sustained and transient language control in the bilingual brain. NeuroImage.

[51] Wang G., Tao L. (2024). Bilingual Language Control in the Brain: Evidence from Structural and Effective Functional Brain Connectivity. J. Cogn. Neurosci..

[52] Wu Y.J., Chen M., Thierry G., Fu Y., Guo T. (2021). Inhibitory control training reveals a common neurofunctional basis for generic executive functions and language switching in bilinguals. BMC Neurosci..

[53] Yuan Q., Wu J., Zhang M., Zhang Z., Chen M., Ding G. (2021). Patterns and networks of language control in bilingual language production. Brain Struct. Funct..

[54] Zhang Y., Huang P., Song Z., Fang L., Shen T., Li Y., Gong Q., Xie P. (2014). In-context language control with production tasks in bilinguals: An fMRI study. Brain Res..

[55] Zhang Y., Wang T., Huang P., Li D., Qiu J., Shen T., Xie P. (2015). Free language selection in the bilingual brain: An event-related fMRI study. Sci. Rep..

[56] Turkeltaub P.E., Eickhoff S.B., Laird A.R., Fox M., Fox P. (2012). Minimizing within-experiment and within-group effects in activation likelihood estimation meta-analyses. Hum. Brain Mapp..

[57] Eickhoff S.B., Laird A.R., Grefkes C. (2009). Coordinate-based activation likelihood estimation meta-analysis of neuroimaging data: A random-effects approach based on empirical estimates of spatial uncertainty. Hum. Brain Mapp..

[58] Lancaster J.L., Tordesillas-Gutiérrez D., Martinez M., Salinas F., Evans A., Zilles K., Mazziotta J.C., Fox P.T. (2007). Bias between MNI and Talairach coordinates analyzed using the ICBM-152 brain template. Hum. Brain Mapp..

[59] Eickhoff S.B., Nichols T.E., Laird A.R., Hoffstaedter F., Amunts K., Fox P.T., Bzdok D., Eickhoff C.R. (2016). Behavior, sensitivity, and power of activation likelihood estimation characterized by massive empirical simulation. Neuroimage.

[60] Müller V.I., Cieslik E.C., Laird A.R., Fox P.T., Radua J., Mataix-Cols D., Tench C.R., Yarkoni T., Nichols T.E., Turkeltaub P.E. (2018). Ten simple rules for neuroimaging meta-analysis. Neurosci. Biobehav. Rev..

[61] Brass M., Von Cramon D.Y. (2002). The role of the frontal cortex in task preparation. Cereb. Cortex.

[62] Abe M., Hanakawa T., Takayama Y., Kuroki C., Ogawa S., Fukuyama H. (2007). Functional coupling of human prefrontal and premotor areas during cognitive manipulation. J. Neurosci..

[63] Bookheimer S. (2002). Functional MRI of language: New approaches to understanding the cortical organization of semantic processing. Annu. Rev. Neurosci..

[64] Cone N.E., Burman D.D., Bitan T., Bolger D.J., Booth J.R. (2008). Developmental changes in brain regions involved in phonological and orthographic processing during spoken language processing. Neuroimage.

[65] Poldrack R.A., Prabhakaran V., Seger C.A., Gabrieli J.D. (1999). Striatal activation during acquisition of a cognitive skill. Neuropsychology.

[66] Bourguignon N.J., Gracco V.L. (2019). A dual architecture for the cognitive control of language: Evidence from functional imaging and language production. NeuroImage.

[67] Kroll J.F., Bobb S.C., Wodniecka Z. (2006). Language selectivity is the exception, not the rule: Arguments against a fixed locus of language selection in bilingual speech. Biling. Lang. Cogn..

[68] Botvinick M.M., Braver T.S., Barch D.M., Carter C.S., Cohen J.D. (2001). Conflict monitoring and cognitive control. Psychol. Rev..

[69] Kerns J.G., Cohen J.D., MacDonald III A.W., Cho R.Y., Stenger V.A., Carter C.S. (2004). Anterior cingulate conflict monitoring and adjustments in control. Science.

[70] Curtis C.E., D’Esposito M. (2003). Persistent activity in the prefrontal cortex during working memory. Trends Cogn. Sci..

[71] Jurado M.B., Rosselli M. (2007). The elusive nature of executive functions: A review of our current understanding. Neuropsychol. Rev..

[72] Sierpowska J., Fernandez-Coello A., Gomez-Andres A., Camins À., Castaner S., Juncadella M., Gabarrós A., Rodríguez-Fornells A. (2018). Involvement of the middle frontal gyrus in language switching as revealed by electrical stimulation mapping and functional magnetic resonance imaging in bilingual brain tumor patients. Cortex.

[73] Swick D., Ashley V., Turken A.U. (2008). Left inferior frontal gyrus is critical for response inhibition. BMC Neurosci..

[74] Moss H.E., Abdallah S., Fletcher P., Bright P., Pilgrim L., Acres K., Tyler L.K. (2005). Selecting among competing alternatives: Selection and retrieval in the left inferior frontal gyrus. Cereb. Cortex.

[75] Zhang J.X., Feng C.M., Fox P.T., Gao J.H., Tan L.H. (2004). Is left inferior frontal gyrus a general mechanism for selection?. NeuroImage.

[76] Ali N., Green D.W., Kherif F., Devlin J.T., Price C.J. (2010). The role of the left head of caudate in suppressing irrelevant words. J. Cogn. Neurosci..

[77] Rodriguez-Fornells A., Rotte M., Heinze H.J., Nösselt T., Münte T.F. (2002). Brain potential and functional MRI evidence for how to handle two languages with one brain. Nature.

[78] DiGirolamo G.J., Kramer A.F., Barad V., Cepeda N.J., Weissman D.H., Milham M.P., Wszalek T.M., Cohen N.J., Banich M.T., Webb A. (2001). General and task-specific frontal lobe recruitment in older adults during executive processes: A fMRI investigation of task-switching. Neuroreport.

[79] Lungu O.V., Binenstock M.M., Pline M.A., Yeaton J.R., Carey J.R. (2007). Neural changes in control implementation of a continuous task. J. Neurosci..

[80] Bunge S.A., Dudukovic N.M., Thomason M.E., Vaidya C.J., Gabrieli J.D. (2002). Immature frontal lobe contributions to cognitive control in children: Evidence from fMRI. Neuron.

[81] Cavanna A.E., Trimble M.R. (2006). The precuneus: A review of its functional anatomy and behavioural correlates. Brain.

[82] Lundstrom B.N., Ingvar M., Petersson K.M. (2005). The role of precuneus and left inferior frontal cortex during source memory episodic retrieval. Neuroimage.

[83] Baddeley A. (1992). Working memory. Science.

[84] Baddeley A. (2003). Working memory: Looking back and looking forward. Nat. Rev. Neurosci..

[85] Jung R.E., Haier R.J. (2007). The Parieto-Frontal Integration Theory (P-FIT) of intelligence: Converging neuroimaging evidence. Behav. Brain Sci..

[86] Bowren M.J., Adolphs R., Bruss J., Manzel K., Corbetta M., Tranel D., Boes A.D. (2020). Multivariate Lesion-Behavior Mapping of General Cognitive Ability and Its Psychometric Constituents. J. Neurosci..

[87] Reynolds M.G., Schlöffel S., Peressotti F. (2016). Asymmetric Switch Costs in Numeral Naming and Number Word Reading: Implications for Models of Bilingual Language Production. Front. Psychol..

