# Supplementary material for: An Activation Likelihood Estimation Meta-Analysis of How Language Balance Impacts the Neural Basis of Bilingual Language Control
Source: Brain Sci. 2025 Jul 28;15(8):803. doi: 10.3390/brainsci15080803 (PMC12384359; doi:10.3390/brainsci15080803)
Supplement: Supplementary file 1 [file brainsci-15-00803-s001.zip › brainsci-3732373-supplementary.pdf]

Supplementary materials

Table S1. A Meta-Analysis of the Language Conversion Studies of Balanced and Unbalanced Bilinguals.

	**Contrast**	**Switch direction**
	Balanced bilinguals (n = 6)	
Garbin [33]	Switch>non-switch	From L2 to L1, from L1 to L2, mixed
Köpke [40]	Switch>baseline	Mixed
Price [41]	Switch>non-switch	Mixed
Reverberi [42]	Switch>non-switch	Mixed
Stasenko [21]	Switch>non-switch	Mixed
Weissberger [36]	Switch>baseline	Mixed
	Unbalanced bilinguals (n = 17)	
Fu [43]	Switch>non-switch	From L2 to L1
Geng [20]	Switch>non-switch	Mixed
Guo [18]	Switch>non-switch	Mixed
Hernandez [44]	Switch>non-switch	Mixed
Hernandez [45]	Switch>non-switch	Mixed
Lehtonen [46]	Switch>non-switch	From L1 to L2
Liu [47]	Switch>non-switch	Mixed
Ma [48]	Switch>baseline	Mixed
Wang [49]	Switch>non-switch	From L2 to L1, from L1 to L2, mixed
Wang [50]	Switch>non-switch	Mixed
Wang [51]	Switch>non-switch	Mixed
Wu [52]	Switch>non-switch	Mixed
Yuan [53]	Switch>non-switch	Mixed
Zhang [54]	Switch>non-switch	Mixed
Zhang [55]	Switch>non-switch	From L2 to L1, mixed
Zhang [37]	Switch>non-switch	Mixed
Zhang [22]	Switch>non-switch	From L2 to L1, from L1 to L2, mixed
